# Effect analysis of the driving factors of super-gentrification using structural equation modeling

**DOI:** 10.1371/journal.pone.0248265

**Published:** 2021-03-12

**Authors:** Jiangang Shi, Kaifeng Duan, Quanwei Xu, Jiajia Li

**Affiliations:** 1 School of Economics and Management, Tongji University, Shanghai, China; 2 Hospitality Management School, Shanghai Business School, Shanghai, China; Institute for Advanced Sustainability Studies, GERMANY

## Abstract

The study of super-gentrification has important practical significance for maintaining social fairness, spatial justice and achieving sustainable urban development. In this article, 23 driving factors influencing super-gentrification are identified by literature research and Delphi method. Then, the 23 driving factors affecting super-gentrification are divided into four dimensions: political, economic, social and spatial dimension. On this basis, hypotheses are proposed and a structural equation model is established. Then, SPSS 25.0 and AMOS 24.0 software are used to test the reliability and validity of the questionnaire data, and the model results are fitted and modified. Finally, the optimization model and path coefficient of super-gentrification driving factors are calculated. The results of the study show that political factors, economic factors, social factors, and spatial factors, all play a positive role in the development of super-gentrification. Social factors are the fundamental factors to promote super-gentrification, political factors, economic factors, and spatial factors also play a key role in the super-gentrification process.

## 1 Introduction

The concept of gentrification was proposed by Glass, a British scholar, in 1964 when describing the succession and replacement of classes in central London [1]. Gentrification is used to describe the phenomenon that the middle class returns to the inner city, attracted by convenient transportation, bustling commerce and specific cultural atmosphere. Gentrification triggered the update of the physical environment and the replacement of social class in the declining inner city [2]. Davidson and Lees (2005) summarize four features of contemporary gentrification: capital re-enters the urban center; the intrusion of high-income groups brings about the upgrading of social class; the change of urban landscape; and the direct or indirect substitution of low-income classes [3].

In recent years, with the emergence of super-gentrification in international metropolises such as New York [4, 5], London [6], Paris [7], and Sydney [8], super-gentrification has become the international frontier of gentrification research. Super-gentrification has played a positive role in preventing urban decline and promoting sustainable urban development.

Super-gentrification is the transformation of already gentrified, prosperous and solidly upper-middle-class neighborhoods into much more exclusive and expensive enclaves. This intensified regentrification is happening in a few select areas of global cities like London and New York that have become the focus of intense investment and conspicuous consumption by a new generation of super-rich ‘financifiers’ fed by fortunes from the global finance and corporate service industries [4].

So far, The most representative and influential research findings on super-gentrification mainly come from Europe and North America. Based on Lees’ seminal paper on super-gentrification, the increase of high-paying jobs in the financial industry on Wall Street and the influx of huge global financial capital have played a significant role in the super-gentrification of Brooklyn Heights [4]. 15 years later, Halasz (2018) conducted another study on the Brooklyn Park Slope, and believed that governmental efforts, the initial gentrification of the area, expansion of employment in lucrative professions, resurgence of urban lifestyles, and growing financialization of real estate are also important driving factors of the super-gentrification in Brooklyn [5]. Butler and Lees (2006) analyzed the super-gentrification in Barnsbury neighborhood in London and concluded that the majority of super-gentrifiers in Barnsbury neighborhood is the product of the elite forms of education (especially Oxford University and Cambridge University) and the globalizing industries of the financial services economy [6]. Morris (2019) conducted a research on the super-gentrification at Millers Point (a downtown area in Sydney, Australia). He argued that government policy and commercialization of urban governance have played an important role in promoting the development of the super-gentrification, and pointed out that, in some cases, super-gentrification could occur in regions which have not experienced gentrification [8]. In the case study of Parkhurst in Johannesburg, South Africa, Monare et al. (2014) found that gender, age and ethnic structure, education and income levels, and the investment demand for real estate are important reasons for super-gentrification [9]. Mendes and Jara’s (2018) research on the Colina de Santana area of Lisbon, the capital of Portugal, found that social polarization, spatial injustice, and the financialization of the real estate industry are the root driving factors of super-gentrification [10]. Rofe (2004) studies Newcastle, a famous steel city in Australia, and found that regional preference and the surge in luxury housing constructions also had a positive effect on super-gentrification [11]. In their research on Houston, Podagrosi et al. (2011) found that government intervention and diversification of urban development investors played a very important role in the process of Houston’s super-gentrification [12]. Zhong et al. (2017) found that uneven distribution of leading state primary and secondary schools and the division of catchment zones are important factors influencing super-gentrification through their research on Wuhan, an important city in central China [13]. Shi et al. (2020) used Interpretive Structural Modeling (ISM) and Matrix Impacts Cross-reference Multiplication Applied to a Classification (MICMAC) to study super-gentrification in Shanghai and found that: housing demand of international elites, investment demand, vigorous development of the real estate industry, and preference for certain areas with superior environment and high-end lifestyle are the fundamental driving factors affecting super-gentrification [14].

To sum up, scholars have made theoretical analysis or empirical research on super-gentrification, these studies provide a rich reference for the further research. However, the current research on super-gentrification mainly focuses on the concept of super-gentrification, while the mechanism of super-gentrification and the effect analysis of driving factors of super-gentrification have not been discussed sufficiently.

In view of this, 23 driving factors affecting super-gentrification were identified based on literature research and Delphi method. Then, the method of structural equation model, which has the functions of causal analysis and path analysis, is adopted to explore the internal structure and influencing mechanism of the driving factors of super-gentrification, hoping to reveal the key influencing factors and their action paths [15]. The research results of this article can enrich the existing gentrification theory, and provide reference for policymakers in formulating policies to promote sustainable urban development.

## 2 Methodology

Structural Equation Modeling (SEM), is an important multivariate linear statistical modeling method. SEM analyzes the relationship between variables based on the covariance matrix of the variables. It has three advantages: processing multiple related dependent variables simultaneously, estimating the structure and relationship of factors simultaneously, and estimating the fitting degree of the model as a whole. SEM allows errors in data estimation and is an important tool for multivariate analysis in various disciplines, especially in economic and social fields. The measurement indicators of super-gentrification degree and its influencing factors all have the characteristics of abstract and multi-dimensional. The variables involved have the characteristics of strong subjectivity, difficult to measure directly, large measurement errors, and complicated causality, especially the explained variables have multiple observational indicators that may be correlated. For the analysis of such a complex system, SEM can give full play to its advantages in the comprehensive application of multiple regression analysis, path analysis and confirmatory factor analysis to analyze the multi-level, complex path and causal path relationship of the system. In this way, method errors caused by traditional statistical analysis can be avoided, and more convincing conclusions can be drawn. Therefore, it is a beneficial exploration to use SEM to study super-gentrification.

The SEM method sets the metric model equation firstly:

X=Λxξ+δ (1)

Y=Λyη+ε (2)

The two equations respectively stipulate the relationship between the result latent variable η and the result observable variable y, and the relationship between the cause latent variable ξ and the cause observable variable x. Λx is the relationship between the cause observable variable and the cause latent variable, and is the factor loading matrix of the cause observable variable on the cause latent variable. Λy is the relationship between the result observable variable and the result latent variable, and is the factor loading matrix of the result observable variable on the result latent variable. δ is the error of the cause observable variable x; ε is the error of the result observable variable y. The structural model equation is set as follows:

η=βη+Γξ+ζ (3)

β is the coefficient matrix of the result latent variable η, and also the path coefficient matrix between the result latent variables; Γ is the coefficient matrix of the cause latent variable ξ, and also the path coefficient matrix of the cause latent variable to the corresponding endogenous latent variable; ζ is the residual term of the structural equation, which is the part failed to explain within the model.

## 3 Driving factors identification and research design

### 3.1 Driving factors identification

Combining the working principle of the structural equation model and the characteristics of the research object, this paper adopts the literature research method and Delphi method to identify the main driving factors of super-gentrification. First of all, we screened out 69 representative literatures [1, 3–13, 16–72] related to the driving factors of super-gentrification, and then used the literature research method to preliminarily determine 28 driving factors influencing super-gentrification. Then, adopting Delphi method, these 28 driving factors were provided to 11 experts from universities and government departments who have rich experience in theory and practice in the field of gentrification. Therefore, they are sufficiently representative and authoritative to determine the key driving factors of super-gentrification. After several rounds of evaluation and feedback from these 11 experts, we finally identified 23 key driving factors affecting super-gentrification. These 23 driving factors of super-gentrification and their meanings are shown in Table 1.

**Table 1:** Driving factors of super-gentrification and their meanings.

Factors	Description	References
S_1_ Economic Globalization	World economic activities cross national boundaries, taking advanced technology and productivity as the means, aiming at maximum profits and economic benefits, and making the world economy an increasingly integrated whole through foreign trade, capital flow, technology transfer and production factors flow.	[1, 4, 6, 13, 16–21]
S_2_ Housing Needs of Overseas Elites	The demand of the well-educated international elite with decent and high-paying jobs to buy and rent houses in city center due to their work and living needs.	[4, 6, 19]
S_3_ Urban Social Stratification	Stratification of city dwellers caused by their differences in income, education, occupation, social status and political rights.	[3, 10, 12, 13, 21–30]
S_4_ Widening Gap between Rich and Poor	The gradual widening of the gap in personal income and wealth within cities, between urban and rural areas, and between industries.	[1, 3, 4, 6, 9, 10, 13, 21, 25, 27, 30–34]
S_5_ Cultural Attraction	The unique material culture and intangible culture such as architecture, history, customs and humanities in a certain region that attract super-gentrifiers to live in this area.	[3, 4, 13, 20, 30, 34–39]
S_6_ Identity Pursuit	Living in the high-end residential area with superior environment, in order to show off their personal taste, wealth and status.	[3–6, 26, 27, 33]
S_7_ Unique Areas and Lifestyle Preferences	Preference of super-gentrifiers for certain region with superior environment and high-end lifestyle in city center.	[1, 4, 5, 11–13, 20, 26, 27, 35, 40]
S_8_ Close to Commercial and Recreational Facilities	There are convenient shopping, cultural, and recreational facilities nearby for super-gentrifiers to consume, relax and entertain.	[4–6, 13, 20, 38, 41–43]
S_9_ Investment Needs	Super-gentrifiers invest in real estate in a specific area of a city in order to prevent their assets from depreciation, and to achieve wealth appreciation.	[4, 6, 9, 27, 34, 44]
S_10_ Demographic Change	Changes in sex ratio, age structure, occupation, education level, and ethnic composition of the population in a specific area and at a certain point of time.	[4, 5, 7, 9, 12, 21, 32, 45–49]
S_11_ Further Improvement of the Market Economy System	Continuous improvement of the Market Economy System in which resources are allocated through perfect competition and free exchange in the market.	[16, 20, 21, 27, 50]
S_12_ Uneven Distribution of Educational Resources and School District Policy	The leading state primary and secondary schools in the city are unevenly distributed, and high-quality educational resources are linked to the house and household registration through the division of catchment zones.	[13, 30, 34, 51, 52]
S_13_ Government Policy Guidance	The government issued a series of regulations and policies to guide the direction of national industrial development, promote the upgrading of industrial structure, coordinate the national industrial structure, and make the national economy develop healthily and sustainably.	[3, 5, 6, 8, 12, 13, 16, 20, 21, 27, 30, 33, 34, 53–60]
S_14_ Commercialization of Urban Governance	The city government operates and manages the city as an enterprise, takes the control of various administrative resources as its capital, to maintain sustainable economic development and steady growth of social wealth, so as to maximize government performance and enhance the competitiveness of the city.	[8, 20, 27, 46, 61–64]
S_15_ Development of the Real Estate Market	Through the construction of new houses and refurbishment of old houses to improve people’s living conditions, while driving the development of numberous related industries, such as finance, construction industry and building materials industry.	[3–5, 10, 11, 20, 21, 37, 38, 54, 65]
S_16_ Popularization of University Education	The increasing proportion of university students in the population aged 18–22.	[5, 6, 66–68]
S_17_ The Rapid Growth of High-Paying Employment Opportunities	High-paying occupations (such as finance, real estate, media, etc.) have increased significantly.	[4–6, 13, 22]
S_18_ Continuous Expansion of Global Financial Capital	Global financial capital is transferred from one country or region to another in pursuit of high speculative profits, that is, the frequent flow of financial capital on a global scale.	[4–6, 17, 33]
S_19_ Early Gentrification in the Region	Gentrification has already occurred in this region, and in this process high-income class invading working class neighborhoods, the landscape of the region and the social class of residents have been improved correspondingly.	[4–7, 11, 13]
S_20_ Re-Urbanization	The re-urbanization of urban centers that have declined due to suburbanization. This process is characterized by an increase in the population of the core urban area and a decrease in the population around the city. At the same time, the city image, urban construction, housing and transportation conditions are improved.	[4, 12, 13, 16, 20, 48, 69–72]
S_21_ Diversity of Urban Development Investors	The main invest body of urban infrastructure construction is gradually shifting from the single state investment to the partnership of the government, private enterprises, private capital and other social capital.	[12, 16, 20, 27]
S_22_ Marketization of Urban Land Use System and Housing System	The use of market mechanism to allocate housing and land resources in order to realize the commercialization and socialization of housing and the full utilization of land resources.	[4, 20, 27, 30, 34, 39, 44, 51, 54]
S_23_ Transformation of Industrial Structure and Occupational Structure in Urban Central Areas	The “suppress the second industry and develop the third industry” in urban central areas, and the occupation structure of the population in the urban center is undergoing a fundamental change.	[4–6, 22, 27, 33]

### 3.2 Research design

#### 3.2.1 Questionnaire design

This article attempts to apply structural equation model to the quantitative study of super-gentrification. The questionnaire consists of two parts. The first part of the questionnaire is a scale for the importance of super-gentrification driving factors, which contains 23 items. According to Likert scale [73], these 23 measurement items, are divided into five levels (Can be ignored, May be important, Important, Very important, Extremely important), corresponding to 1 to 5 points respectively. The second part is the personal information of the respondents, including age group, occupation, relevant work or research years and education background [74, 75].

#### 3.2.2 Data collection

The respondents of this study are experts and scholars in the field of gentrification, PhD and master candidates who have participated in urban renewal research, middle and senior managers of real estate development and agency companies, and private entrepreneur, etc. The basic information of the respondents is shown in Table 2. We explained the purpose of the survey to the participants and ensured that the data received is confidential. The survey lasted about 4 months, from March 2020 to June 2020. The questionnaire survey was distributed by e-mail and Wenjuanxing, which is an online questionnaire survey platform in China. A total of 219 questionnaires were returned, of which 209 were valid, with an effective rate of 95.4%. There is no consensus on the acceptable thresholds for sample sizes among researchers that used SEM. In the book Principles and practice of structural equation modelling, Kline (2010) claims that a sample size of 200 is fair for SEM statistical analysis [76]. A group of researchers even used smaller sample sizes, e.g. Wasiuzzaman et al. (2021) used 169 samples [77]; Coon et al. (2020) 149 used samples [78]; Molwus et al. (2017) used 61 samples [79]. In this study, the inherent difficulty to collect questionnaire data coupled with the characteristics sought in the targeted respondents limit the number of eligible respondents. In addition, the model to be tested using SEM is not overly complex and source of data is very reliable in this study, so the sample size of 209 can be enough [80]. Therefore, the sample size met the requirements of the SEM.

**Table 2:** Personal information of respondents.

Variables	Categories	Frequency	Percentage
Age	21–30	48	23
	31–40	110	52.6
	41–50	37	17.7
	Over 50	14	6.7
Occupation	Scholar	77	36.8
	Ph.D. candidate	27	12.9
	Master candidate	8	3.8
	Middle and senior managers of real estate development and agency companies	65	31
	Government official	10	5
	Private entrepreneur	9	4.3
	Other	13	6.2
Time of work or research related to gentrification	1–3 years	70	33.5
	3–5 years	28	13.4
	5–7 years	26	12.4
	more than 7 years	85	40.7
Education	Junior college degree and below	8	3.8
	Bachelor	29	13.9
	Master	75	35.9
	Ph.D.	97	46.4
Nationality	China	201	96
	Other countries	8	4.0

## 4 Reliability and validity test

### 4.1 Reliability test

Reliability refers to the reliability of the measurement tool or method itself, that is, a reflection of whether the same information can be obtained after repeated observations of the same phenomenon [81]. Reliability mainly examines the stability and consistency of the measured results. Different measurement methods will produce different types of reliability, such as test-retest reliability, alternative-form reliability, split-half reliability, and internal consistency reliability, etc. In this article, Cronbach’s Alpha is used to check the internal consistency reliability of the questionnaire. It is generally believed that Cronbach’s α coefficient value has the following meanings: α>0.9 represents very high reliability; 0.8<α<0.9 represents high reliability; 0.7<α<0.8 represents acceptable reliability, if α < 0.7, it indicates that the reliability is questionable or unacceptable [81].

Reliability Analysis of 209 questionnaires was carried out using the Reliability Analysis tool of IBM SPSS Statistics 25.0 software. The results show that the Cronbach’s α coefficient is 0.926, greater than 0.9, indicating that the reliability of the questionnaires data is ideal [82].

### 4.2 Validity test

Validity refers to the extent to which a measurement tool or method can accurately measure the concept of the study variable. In other words, validity is used to measure whether a certain concept or structure can be truly expressed by the corresponding measurement item, and to what extent it can be measured. In addition, it is also used to measure the structural relationship between variables and items. The more consistent the measurement results with the content to be measured, the higher the validity; otherwise, the lower the validity.

In this article, KMO Test and Bartlett’s Test of Sphericity are used for validity analysis. KMO (Kaiser-Meyer-Olkin) Test is used to compare the relative size of the Pearson correlation coefficient and Pearson correlation coefficient among the original variables. The KMO value is between 0 and 1. The closer the value is to 1, the stronger the correlation between variables and the more suitable for factor analysis; otherwise, it is not suitable for factor analysis [83]. Bartlett’s Test of Sphericity is used to test whether the correlation matrix is an identity matrix, that is, whether each variable is independent. If it is an identity matrix, it indicates that the data is not suitable for factor analysis. In general, the smaller the significance level, the more likely there is a meaningful relationship between the original variables. The test results of this study are shown in Table 3.

**Table 3:** KMO and Bartlett’s test.

Kaiser-Meyer-Olkin Measure of Sampling Adequacy		0.904
Bartlett’s Test of Sphericity	Approx. Chi-Square	2325.435
	df	253
	Sig.	0.000

In Table 3, the KMO value is 0.904, greater than 0.9, indicating that the factor analysis effect is good. The value of Bartlett’s test of sphericity is 2325.435. When the degree of freedom is 253, P<0.05, reaching the significance level. Therefore, this study is suitable for factor analysis.

## 5 Construction of structural equation nodeling

### 5.1 Exploratory factor analysis

SPSS 25.0 is used for correlation analysis of the above structural parameters, and it is found that there is a strong correlation between some structural parameters, so it is not suitable for direct path analysis. To this end, exploratory factor analysis method was used to reduce the dimensions of the parameters (combining strongly correlated parameters) to form several uncorrelated or weakly correlated super-gentrification driving factor dimensions. Then take them as the main influencing factors in path analysis.

According to the results of SPSS 25.0 exploratory factor analysis and combined with professional knowledge, the driving factors that affect super-gentrification are divided into four dimensions, namely: political dimension, economic dimension, social dimension and spatial dimension. Among them, the political dimension is measured by five indicators, namely, Government Policy Guidance, Commercialization of Urban Governance, Development of the Real Estate Market, Diversity of Urban Development Investors, Marketization of Urban Land Use System and Housing System. The economic dimension is measured by seven indicators, namely, Economic Globalization, Housing Needs of Overseas Elites, Investment Needs, Further Improvement of the Market Economy System, The Rapid Growth of High-Paying Employment Opportunities, Continuous Expansion of Global Financial Capital, Transformation of Industrial Structure and Occupational Structure in Urban Central Areas. The social dimension is measured by five indicators, namely, Urban Social Stratification, Widening Gap between Rich and Poor, Demographic Change, Popularization of University Education, Re-Urbanization. The spatial dimension is measured by six indicators, namely, Cultural Attraction, Identity Pursuit, Unique Areas and Lifestyle Preferences, Close to Commercial and Recreational Facilities, Uneven Distribution of Educational Resources and School District Policy, Early Gentrification in the Region.

### 5.2 Theoretical analysis and hypotheses

● The relationship between the driving factors of political dimension and super-gentrification

In China, the government is the sole legal owner of urban land. The government plays an important role in the process of sorting out fragmented land ownership, demolition and resettlement, land clearing, land transfer, promoting capital circulation, stimulating middle class demand, and real estate sales [20, 33].

With the market-oriented transformation of land use system and housing system, the governments of major cities in China set off a wave of urban renewal at the end of the 20th century. Urban renewal has significantly improved the urban environmental landscape. The urban center area, after renovation and gentrification, has attracted the concentration of the super-gentrifiers and high-end service industries. The super-gentrifiers began to “invade” the former gentrification neighborhoods [4, 27].

Therefore, the advancement of the housing system reform provides a market-oriented foundation and policy guarantee for super-gentrification [27, 39]; the acceleration of urban renewal creates good opportunities and material conditions for super-gentrification [16, 71]. The driving factors of political dimension are undoubtedly very important driving factors for the emergence of super-gentrification.

Therefore, this study proposes the following hypothesis:

H1: The driving factors of political dimension have positive correlation with super-gentrification.
● The relationship between the driving factors of economic dimension and super-gentrification

Economic globalization has accelerated the pace of urban industrial structure transformation [6, 19]. High-end information industry, finance and insurance, commerce and entertainment, and real estate have gradually replaced low-end traditional industries in urban centers [4–6]. At the same time, the continuous expansion of global capital enables a large amount of foreign capital to directly participate in the process of urban renewal and real estate development, which greatly promotes the reconstruction of urban social space [17, 27].

As an important type of gentrification, super-gentrification is a process of social space reconstruction of urban function transformation and consumption upgrading. Since the beginning of the 21st century, with the rapid development of economy and society, cities are transforming from homogeneous to heterogeneous and from production-led to consumption-led. The globalization of capital flow, the rise of super-gentrifiers, and the market-oriented reform of real estate, give rise to the phenomenon of continuous upgrading and differentiation of social stratum, material space, residents’ consumption, and cultural taste within cities. All these have created favorable conditions for the emergence of super-gentrification in Chinese cities.

Therefore, this study proposes the following hypothesis:

H2: The driving factors of economic dimension have positive correlation with super-gentrification.
● The relationship between the driving factors of social dimension and super-gentrification

With the profound changes that have taken place in Chinese society, urban social stratification has become increasingly obvious [22, 23], the wealth gap between the rich and the poor has gradually widened [27, 32], the trend of social polarization has begun to emerge. The differences in social status, economic capacity, and value orientation among classes has become increasingly obvious [26, 29]. Wind data shows that from 2003 to 2017, China’s Gini coefficient was higher than 0.46. Internationally, 0.4 is commonly regarded as the warning line for the gap between the rich and the poor. In the process of differentiation and reconstruction of the social structure during the transition period, the emergence and growth of the super-gentrifiers is an indisputable fact. Although the size of the super-gentrifiers is still very limited nationwide, it has already accounts for a considerable proportion in some megacities.

In the context of housing market-oriented reform and large-scale urban renewal, a large number of traditional houses in Chinese cities have been demolished and rebuilt or transformed into high-end gated communities [21]. Compared with other classes, super-gentrifiers with higher income level show stronger ability to choose residence, and are in an active and advantageous position in the competition for residential areas in the central city. In this way, the indigenous residents in the central city are replaced by the super-gentrifiers with higher income.

Therefore, this study proposes the following hypothesis:

H3: The driving factors of social dimension have positive correlation with super-gentrification.
● The relationship between the driving factors of spatial dimension and super-gentrification

In the course of historical evolution, cities will gradually form a certain social space structure. The affluent classes usually live in areas with superior living environments, and these areas are often regarded as the high-end residential areas in the city. And this kind of social space structure has certain temporal and spatial stability and continuity [5, 27, 84].

As Western consumer culture gradually spreads into China, the wealthy super-gentrifiers has begun to advocate conspicuous consumption with symbolic value. They favor high-end, fashionable and international consumption patterns that can better express their personality and taste, highlight their identity and status, and reflect their lifestyle and self-worth, indicating the lifestyle, cultural concepts, habits and tastes corresponding to individuals or groups of different classes [5, 26, 33]. Housing is an important property and status symbol of residents. Residents’ occupational status, economic income and political capital are important factors affecting housing acquisition. The acquisition of housing resources is a comprehensive manifestation of an individual’s possession of resources and ability, which can be used to measure the social class in which they are located, and is an important indicator of an individual’s social status [26, 33].

Super-gentrification is a process in which super-gentrifiers with strong economic strength, originally scattered in the city or newly immigrated to the city, gather in the high-end urban residential areas by purchasing and migrating to new houses. Due to the historical inertia of urban development, urban development in each period is basically the inheritance and development of urban spatial structure of the previous period [43]. Therefore, the social space image of high-end residential area will affect residents’ behavior in choosing residence to a certain extent.

Therefore, this study proposes the following hypothesis:

H4: The driving factors of spatial dimension have positive correlation with super-gentrification.

### 5.3 Initial model construction

The relationship between the influencing factors of super-gentrification studied in this article is an issue that cannot be observed directly but is to be studied. Structural equation model reflects the latent variables that are difficult to measure through some directly observable variables. This model can deal with multiple dependent variables at simultaneously and allows independent variables and dependent variables with error terms. At the same time, the structural relationships between the factors are estimated. Therefore, this article uses structural equation model to analyze the mechanism of action and conduction path of each factor on super-gentrification through path analysis and effect size analysis.

According to the results of SPSS 25.0 exploratory factor analysis and research hypotheses, the driving factors of policy, economy, society and space all have significant impact on super-gentrification. Based on this, this study constructs a theoretical model of structural relationship of the driving factors of super-gentrification. The endogenous latent variable of this model is super-gentrification, the exogenous latent variables are political factors, economic factors, social factors, and spatial factors, and the Observed Variables include 23 factors such as Government Policy Guidance, Economic Globalization, Demographic Change, Close to Commercial and Recreational Facilities. The specific names and symbols of the variables are shown in Table 4 [85].

**Table 4:** Variables summary of structural equation model.

Latent Variables	Constructs	Observed Variables	Items	Error
Exogenous Latent Variables	Political Factors	Government Policy Guidance	X11	e11
		Commercialization of Urban Governance	X12	e12
		Development of the Real Estate Market	X13	e13
		Diversity of Urban Development Investors	X14	e14
		Marketization of Urban Land Use System and Housing System	X15	e15
	Economic Factors	Economic Globalization	X21	e21
		Housing Needs of Overseas Elites	X22	e22
		Investment Needs	X23	e23
		Further Improvement of the Market Economy System	X24	e24
		The Rapid Growth of High-Paying Employment Opportunities	X25	e25
		Continuous Expansion of Global Financial Capital	X26	e26
		Transformation of Industrial Structure and Occupational Structure in Urban Central Areas	X27	e27
	Social Factors	Urban Social Stratification	X31	e31
		Widening Gap between Rich and Poor	X32	e32
		Demographic Change	X33	e33
		Popularization of University Education	X34	e34
		Re-Urbanization	X35	e35
	Spatial Factors	Cultural Attraction	X41	e41
		Identity Pursuit	X42	e42
		Unique Areas and Lifestyle Preferences	X43	e43
		Close to Commercial and Recreational Facilities	X44	e44
		Uneven Distribution of Educational Resources and School District Policy	X45	e45
		Early Gentrification in the Region	X46	e46
Endogenous Latent Variable	Super-gentrification			

**Fig 1:**
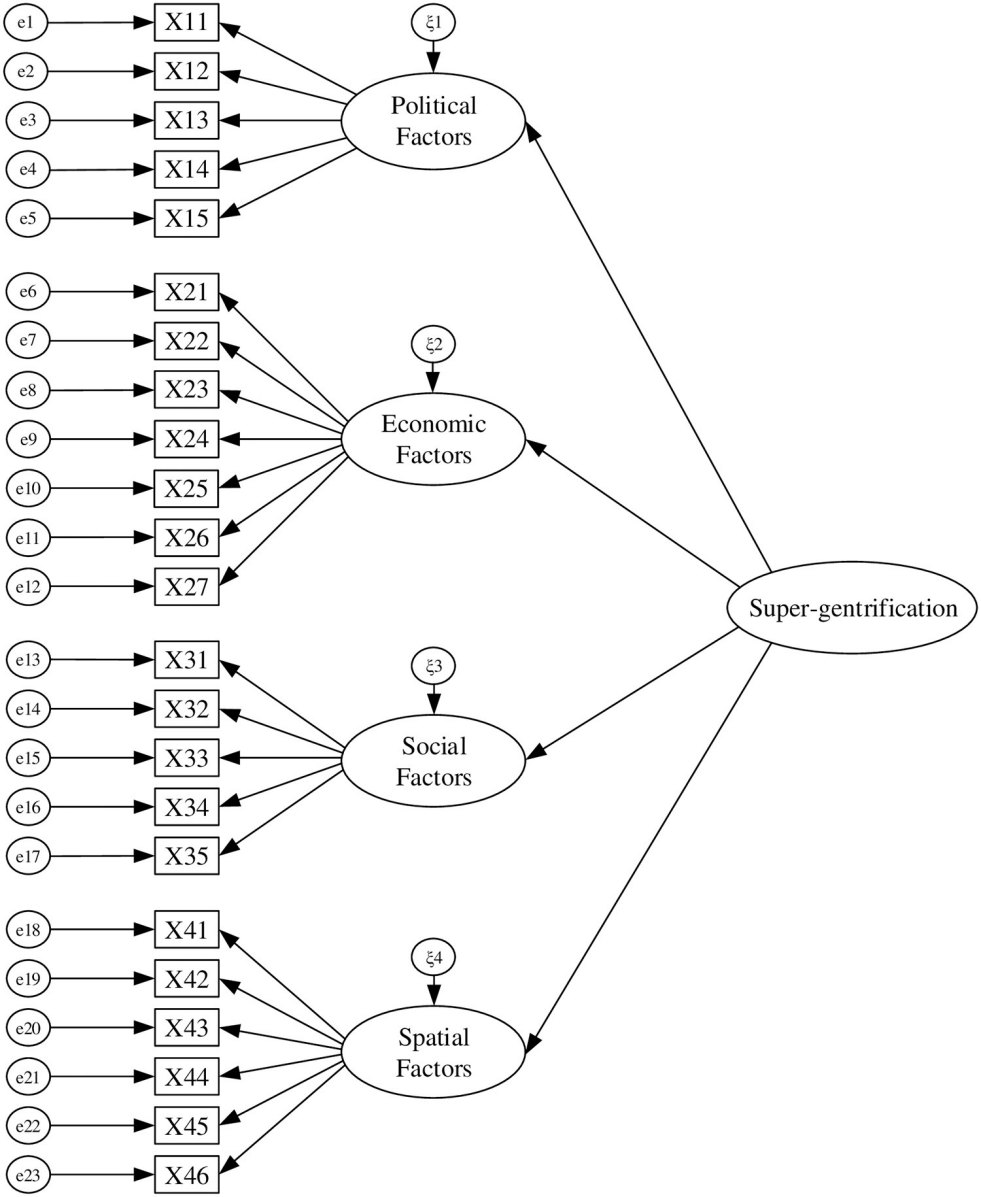
Initial structural equation model.

Based on the previous theoretical analysis and assumptions, an initial structural equation model of the relationship between driving factors and super-gentrification is established, as shown in Fig 1.

## 6 Initial model fitting and model modification

### 6.1 Initial model fitting

The fitting effect of structural equation model indicates whether the interaction between variables exists. By testing the fitting effect of the model, the model is continuously optimized until the model with the best fitting effect is found, so as to prove whether the correlation between variables exists.

The commonly used goodness-of-fit indices of structural equation model are as follows: $\frac{\chi^{2}}{df}$, Ratio of chi-square to degree of freedom; RMESA, Root Mean Square Error of Approximation; RMR, Root Mean square Residual; GFI, Goodness of Fit Index; NFI, Normed Fit Index; CFI, Comparative Fit Index; IFI, Incremental Fit Index. Their criteria are shown in Table 5 [86].

**Table 5:** Fit summary of criteria and initial model.

Fit Indices	Absolute Fit Indices				Relative Fit Indices		
	$\frac{\chi^{2}}{df}$	GFI	RMR	RMSEA	NFI	IFI	CFI
Criteria	<3	>0.90	<0.08	<0.06	>0.90	>0.90	>0.90
Initial Model	2.774	0.785	0.086	0.092	0.741	0.818	0.815

In this study, AMOS 24.0 software is used to conduct the initial model fitting test of the structural equation. The ideal value range of the fitting Indices and the fitting value of initial model are shown in Table 5.

As can be seen from Table 5, the values of all fitting indexes of the initial model are not ideal except $\frac{\chi^{2}}{df}$, and the fitting effect of the initial model is not good. Therefore, the initial model needs to be modified.

### 6.2 Model modification

There are two main purposes to modify the model. One is to make $\frac{\chi^{2}}{df}$ less than 3, that is, to make the model fit the data as much as possible; the other is to make each goodness of fit index approach or meet the requirements of the standard value.

The specific modification steps are as follows:

In the first step, according to the fitting results of the initial structural equation model by AMOS 24.0 software, the factor loading of the item “Government Policy Guidance” is 0.48, which is deleted because it does not meet the ideal standard of 0.5.

In the second step, using the Modification Indices (MI) option in AMOS 24.0 software to find the path with the maximum MI value and add the path, that is, the initial model is modified once.

The third step is to run the AMOS 24.0 software to obtain the goodness of fit index value of the modified model to verify the fitting effect of the modified model.

Repeat the above steps until the value of the goodness of fit index of the modified model approaches or meets the requirements of the standard value.

**Fig 2:**
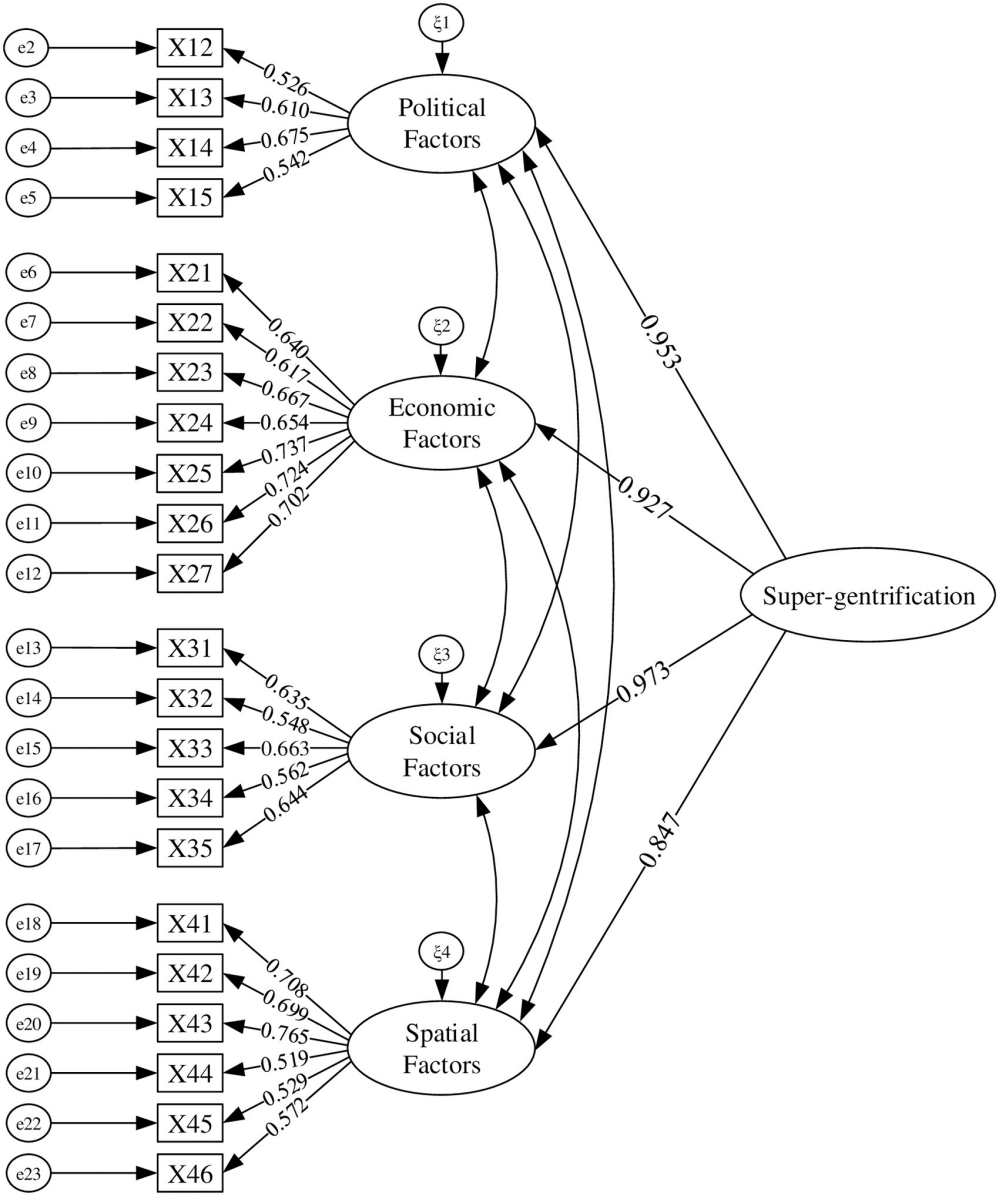
Best modified structural equation model.

According to the above method, the initial model is modified several times, and finally the optimal structural equation model is obtained, as shown in Fig 2.

Compared with the initial model, the modified structural equation model has better fitting effect. The values of the goodness of fit index of the modified structural equation model are shown in Table 6.

**Table 6:** Fit summary of best modified model.

Fit Indices	Absolute Fit Indices				Relative Fit Indices		
	$\frac{\chi^{2}}{df}$	GFI	RMR	RMSEA	NFI	IFI	CFI
Criteria	<3	>0.90	<0.08	<0.06	>0.90	>0.90	>0.90
Modified Model	1.325	0.912	0.058	0.040	0.903	0.974	0.974

According to the summary of goodness of fit index in Table 6, the fitting of the final model after modification can be known. The values of $\frac{\chi^{2}}{df}$, RMSEA, RMR, GFI, NFI, IFI and CFI all meet the fitting criteria.

On the whole, most of the fit indices of the modified model have been greatly improved. The fit degree of the model is relatively good and basically passes the test of goodness of fit. In other words, the correlation between variables in the modified structural equation model shown in Fig 2 does exist.

The path coefficient and hypothesis testing results of the theoretical model are shown in Table 7. According to the data in the Table 7, the P values of the expected hypotheses H1, H2, H3, and H4 are all less than 0.05, indicating that these hypotheses are supported by the data.

**Table 7:** Hypotheses testing results.

Hypothesis	Path Correlation	Path Coefficient	P	Results
H1	Political Factors→Super-gentrification	0.953	***	Supported
H2	Economic Factors→Super-gentrification	0.927	***	Supported
H3	Social Factors→Super-gentrification	0.973	***	Supported
H4	Spatial Factors→Super-gentrification	0.847	***	Supported

Note:

***means P<0.001.

## 7 Conclusion and discussion

On the basis of studying the influence mechanism theory of super-gentrification, this article discusses the influence of political factors, economic factors, social factors, and spatial factors on the process of super-gentrification. According to the test results of the structural equation model, the influence degree of these factors on the process of super-gentrification is obtained.

The research results show that political factors, economic factors, social factors, and spatial factors all play a positive role in the development of super-gentrification. The mean values of the four driving factors dimensions of super-gentrification are, in descending order, as follows: social factors: 3.422; political factors: 3.406; spatial factors: 3.355; economic factors: 3.241. The mean value of social factors is the largest, and the mean value of economic factors is the smallest. The path coefficients of the 4 exogenous latent variables in the structural equation model are all positive. The values in descending order are: social factors: 0.973; political factors: 0.953; economic factors: 0.927; spatial factors: 0.847. Therefore, the hypotheses H1, H2, H3 and H4 proposed in this article are all supported.

Chinese society is facing a situation in which the gap between the rich and the poor has widened and the polarization of social classes has emerged in urban development. China’s current class distribution is still far from the olive-shaped social structure in which the middle class is fully developed. China’s housing system reform has enabled people to pursue housing conditions according to their own wishes and economic capabilities. The high-quality residential areas in urban center are gradually being replaced by younger, higher-income, and better-educated super-gentrifiers with decent jobs, forming a super-gentrifiers settlement area. Meanwhile, the super-gentrifiers also hope to realize others’ recognition of their social status through specific consumption patterns or pursuit of living conditions, such as living in high-end communities with perfect infrastructure and social service facilities, and conducting high-end consumption. Therefore, the social factors play a leading role in the process of super-gentrification.

The housing, tax and credit policies formulated by the government provide impetus for the development of super-gentrification. The marketization of the urban land use system makes land transfer fees an important financial source for local governments. Under the guidance of GDP only, “commercialized” local governments have an urgent need for economic growth. Meanwhile, with the development of the real estate market, real estate enterprises and investment companies have become important forces to promote economic development. In order to maximize local fiscal revenue and political performance, the local government, which has monopolistic administrative resources such as urban planning and land transfer, and real estate enterprises pursuing profit maximization formed an “Urban Growth Alliance” to jointly transform and reshape the central area of the city. Therefore, the government has largely guided and promoted super-gentrification through land management and urban planning policies.

In the context of economic globalization and further improvement of the market economy system, many internationally renowned multinational companies and financial institutions have gathered in the city center of Shanghai, making Shanghai one of the cities that accepts the most international capital in the Asia-Pacific region. As a result, the number of foreign elites who come to work and live in Shanghai has increased significantly. Great changes have taken place in the industrial structure and occupational structure in the urban center. The secondary industry and the low-level service industry in the tertiary industry in the urban center are gradually being replaced by the finance, insurance, and information industries with high knowledge and technology. In addition, the huge appreciation potential of the real estate in the central city has attracted a large amount of investment and speculative funds constantly flowing into the real estate market in the urban center. Therefore, the economic factors are powerful driving force in the process of super-gentrification of urban center.

Super-gentrification is a cultural movement driven by unique areas and lifestyle preferences. The naturally formed streets, historical buildings, and socio-cultural diversity in the inner city area have a huge attraction to the super-gentrifiers. They move to the inner city area in order to satisfy their own aesthetic and cultural needs. In addition, proximity to commercial, recreational and cultural facilities is also an important reason why the super-gentrifiers choose to return to the inner city area. Besides, some super-gentrifiers parents choose to move to the inner-city districts with high quality primary and secondary school admission qualifications for the sake of their children can get a good education. Therefore, the spatial factors play an important pulling role in the process of super-gentrification.

In summary, social factors are the fundamental factors that promote super-gentrification. Political factors, economic factors and spatial factors also play a key role in the process of super-gentrification. Due to its positive impact on urban sustainable development, gentrification has gradually become a "global urban strategy". When the decision-making authority formulates and implements the urban renewal strategy, it is recommended that relevant policies and incentive measures be adopted according to the path relationship shown in the structural model, avoiding the negative effects such as social polarization and depriving the interests of vulnerable groups in the process of super-gentrification.

Based on previous research results, this article constructs a structural equation model for the driving factors of super-gentrification. Although this model shows ideal reliability and validity, its suitability in the study of super-gentrification remains to be further explored.

## Supporting information

S1 Data (XLSX)
